# Being mothers and fathers of a child with type 1 diabetes aged 1 to 7 years: a phenomenological study of parents’ experiences

**DOI:** 10.1080/17482631.2018.1487758

**Published:** 2018-06-26

**Authors:** Anne Solveig Iversen, Marit Graue, Anne Haugstvedt, Målfrid Råheim

**Affiliations:** a Department of Nursing, Faculty of Health- and Social Sciences, Western Norway University of Applied Sciences, Bergen, Norway; b Centre for Evidence-Based Practice, Faculty of Health- and Social Sciences, Western Norway University of Applied Sciences, Bergen, Norway; c Department of Global Health and Primary Care, University of Bergen, Bergen, Norway

**Keywords:** Diabetes type 1, parenting, chronic illness, children, phenomenology

## Abstract

**Purpose**: The diagnosis of diabetes in pre-school children poses a number of unique challenges related to everyday responsibility, and the continuous need for supervision and caregiving. This may affect both the child’s and the parents’ perceived burden caused by the condition. The aim of the study was to explore the lived experience of being mothers and fathers of a child with type 1 diabetes aged 1 to 7 years.

**Methods**: The study is rooted in an interpretive phenomenological methodology as described by van Manen. In-depth interviews were carried out to collect data.

**Findings**. We were able to identify one essential theme across the interviews: *Striving to live an ordinary family life, yet feeling and living very differently*—with interrelated sub-themes: *A life-changing situation, Always on guard*, and *Struggling to let go*.

**Conclusion**: Parents described a profoundly changed situation, and they were indeed striving to live like a “normal” family. They were in need of support from health care professionals at the outpatient clinic, not only support and supervision in regard to practical tasks, but also concerning handling a changed life situation and emotional reactions, especially in the first year after diagnosis.

## Introduction

Type 1 diabetes (T1D) is one of the most common chronic conditions among children. In 2015, estimates from the International Diabetes Federation indicated that approximately 86.000 children <15 years of age develop T1D worldwide every year (International Diabetes Federation, 2015). The incidence of childhood T1D has increased in the last few decades with the highest increase in incidence among pre-school children (Patterson et al., 2009). Norway is among the countries with the highest incidence rates of childhood T1D, with an incidence of 36.5 per 100,000 person-years in children < 15 years in 2015 (Skrivarhaug, Kummernes, & Drivvoll, 2016). Among a totality of 329 children diagnosed with diabetes in Norway in 2016, the incidence of children < 5 years of age was 68. The main goals of diabetes treatment among children with T1D are to achieve the best feasible blood glucose concentrations, to minimise the occurrence of hypoglycaemic events, and to prevent long-term complications (Rewers et al., 2014). Thus, the treatment of children with T1D requires multiple medical decisions and technical procedures related to blood glucose monitoring and insulin administration, every day. The diagnosis of diabetes in pre-school children poses a number of unique challenges upon the parents. Thus, it is important to gain more knowledge from the parents’ perspective on treatment issues as well as the everyday challenges parenting a small child with diabetes (Sullivan-Bolyai, Deatrick, Gruppuso, Tamborlane, & Grey, 2003). The challenges are related to among others physical growth, day—and night-time monitoring, insulin dose adjustments, changing food preferences, irregular physical activity patterns, difficulty in describing and communicating their symptoms clearly, and the continuous need for supervision and caregiving (Streisand & Monaghan, 2014). These challenges may affect both the child and the parents’ perceived burden of the disease. Mothers of young children with T1D describe a need to maintain constant vigilance and alertness related to the child’s diabetes (Sullivan-Bolyai et al., 2003; Sullivan-Bolyai, Rosenberg, & Bayard, 2006).

The International Society for Pediatric and Adolescent Diabetes (ISPAD) guidelines emphasises the importance of family factors for the management of diabetes in children (Acerini, Craig, De Beaufort, Maahs, & Hanas, 2014). Several studies have emphasised parental psychological distress when and immediately after a child is diagnosed with T1D (Landolt, Vollrath, Laimbacher, Gnehm, & Sennhauser, 2005; Lowes & Lyne, 2000; Stallwood, 2005; Streisand et al., 2008). Symptoms such as shock and sorrow (Lowes & Lyne, 2000), post-traumatic stress disorder (Landolt et al., 2005) and increased anxiety and depression levels (Streisand et al., 2008) are common among parents of children newly diagnosed with diabetes. To learn to give your own child injections and finger pricks could be both a practical and an emotional challenge for parents. In a systematic review, Whittemore, Jaser, Chao, Jang, and Grey (2012) revealed a prevalence of parental psychological distress at diagnosis of between 20% and 30%. Parents have described a feeling of being overwhelmed by the management demands, a feeling that persisted over time when experiencing the complexity of daily management, the constant fear of especially hypoglycaemic events and of new situations coming up (Smaldone & Ritholz, 2011; Sullivan-Bolyai et al., 2003; Wennick & Hallström, 2006). The fear of nighttime hypoglycaemia has been reported as extra challenging among parents of the youngest children (Monaghan, Hilliard, Cogen, & Streisand, 2009). In a previous Norwegian study among children up to 15 years of age and their parents, a higher frequency of blood glucose measurements and a more optimal glycaemic control was identified among the children in the youngest age group (1–5 years) compared to the age groups from 6 years and above (Haugstvedt, Rokne, Graue, & Wentzel-Larsen, 2011). The parents’ levels of perceived burden and fear of hypoglycaemia were, however, not associated with the child’s age (Haugstvedt, Wentzel-Larsen, Rokne, Graue et al., 2011, Haugstvedt, Wentzel‐Larsen, Graue, Søvik, & Rokne, 2010), but an association between better glycaemic control in children and maternal perceived social limitation was revealed. This might indicate that mothers who spend much of their total available time and energy on treatment issues perceive this as incredibly demanding (Haugstvedt, Rokne et al., 2011). However, the perceived lack of freedom might also be related to difficulties in transferring responsibility for the diabetes management to other people. Kindergarten entry represents normally the first separation from parents after the diagnosis, and requires that personnel are attentive and competent in this meeting (Smaldone & Ritholz, 2011). Situations that undermine the trust as well as situations that strengthens the trust are critical.

## The study

### Aim

The aim of this study was to explore the lived experience of being mothers and fathers of a young child with T1D aged 1 to 7 years, who had had the diagnosis for at least 1 year.

### Methodology and method

The study is rooted in an interpretive phenomenological methodology as described by van Manen (Van Manen, 1997, 2006). The grounding in phenomenology means that it seeks to gain in-depth understanding of particular phenomena in the life world by giving attention to the experience and perceptions of those who live them through (ibid), in our case lived experience of mothers and fathers of a young child with T1D. Researching the lived experience of other persons challenges the researcher´s pre-understanding fundamentally, and demands her to take a phenomenological attitude. According to van Manen (Sævi, 2013) and Dahlberg, Dahlberg, and Nyström (2008), a phenomenological attitude means to make explicit understandings and beliefs, and to purposefully seek an attitude of openness, wonder and thoughtfulness in the process of trying to understand another person. Thus, pre-understanding is both a prerequisite for and a possible obstacle to new understanding, and the researcher has to consciously cultivate a phenomenological attitude. According to Heidegger (1962), understanding each other is never neutral or fully distanced, but engaged and in need of sensitivity (Vetlesen & Stänicke, 1999), and part of the phenomenological attitude is to be sensitive towards the other´s lived experience.

### Participants

The participants were recruited from a paediatric outpatient clinic in Norway. Mothers and fathers of children aged 1 to 7 years who had been diagnosed with T1D for at least 1 year were eligible for participation. Head Nurses of the wards invited the participants into the study. A diabetes nurse at the outpatient clinic further informed eligible parents about the project during a follow-up consultation. A total of 18 mothers and fathers were invited. Of them, eight mothers (mean age 30 years, range 26–40 years), and seven fathers (mean age 38 years, range 29–46 years) gave their consent to participate (Table I). These 15 participants consisted of seven couples and one mother (without the father). In some families, a sibling as well as one of the parents also had T1D. One child had coeliac disease in addition to the T1D.

**Table I.** Participant characteristics.

Parents	Employment status	Children’s age at diagnosis	Children’s diabetes duration	Diabetes treatment
Mother child 1	100%	2	1	Pen
Father child 1	100%	2	1	Pen
Mother child 2	80%	4	2	Pump
Mother child 3	60%	1	6	Pump
Father child 3	100%	1	6	Pump
Mother child 4	60%	2	1	Pump
Father child 4	100%	2	1	Pump
Mother child 5	80%	3	2	Pump
Father child 5	100%	3	2	Pump
Mother child 6	80%	3	2	Pump
Father child 6	100%	3	2	Pump
Mother child 7	80%	2	2	Pump
Father child 7	80%	2	2	Pump
Mother child 8	80%	5	2	Pump
Father child 8	100%	5	2	Pump

### Data collection

After giving signed informed consent, the Head Nurses sent contact information to the nurse who conducted the interviews (the first author). She made appointments with the parents. In-depth interviews were carried out in 2015. All parents gave written consent before the interviews. Of the 15 participants, 13 were interviewed separately, and one couple were interviewed together. All the interviews were conducted in a place chosen by the parents. Seven interviews were conducted face-to-face in the parents’ homes, four interviews in the office of the nurse, two interviews in a silent corner in a café, and one at the participant’s workplace. One of the parents preferred to be interviewed by telephone. The interview guide had open questions such as: “How did you experience the time around getting the diagnosis?” “How did you experience the daily burdens as a mother/father of a child with T1D?” The interviews lasted from 45–90 minutes.

### Ethical considerations

The study was presented for the Western Norway Regional Committee for Medical and Health Research Ethics, and approved by the Norwegian Data Inspectorate (Project no 2013–35,755), and performed in accordance with the Helsinki Declaration. Relevant hospital authorities approved the study as well. Anonymity, informed written consent and voluntary participation were important ethical prerequisites. In-depth interviews require that the researcher is sensitive, especially when the interview topics may touch deeply rooted worries, suffering, and challenges in peoples’ lives, as in our case. In the conversation with the parents, the interviewer was aware of the parents’ vulnerability. The parents were informed that they could withdraw from the study at any time without any consequences for the child´s treatment.

### Data analysis

The first author transcribed the in-depth interviews verbatim, and wrote down the themes arising when reading and rereading the interview text as a whole. The analysis involved holistic reading with a thematic dimension of enquiry, and asking: “What does this text speak about?” as recommended by Van Manen (1997, p.345). Preliminary identification and reflection on themes and their meanings took place for each interview and before analysis across the interview texts. Analysis across interview texts meant to identify and reflect on essential themes arising from the texts. After reading single interviews, we as a group of female researchers discussed the text, asking several times what the main message was. It is necessary to determine the themes around which the description will be woven (Van Manen, 1997, p.106), and to differentiate between essential themes and sub-themes. The co-authors were involved in holistic reading in all (M.R) and parts (M.G., A.H.) of the interview material, and discussing what essential meanings the text spoke about. The analysis moved back and forth between the essential theme and sub-themes until consensus were reached. When writing the article, all took part. The last author (M.R.) was more involved than the second and third author in the analysis and writing up the essential themes.

## Findings

The sample consisted of 15 interviews with parents, eight mothers (26–40 years) and seven fathers (29–46 years) of a child with T1D (aged 1 to 7 years). We were able to identify one essential theme across the interviews, consisting of three sub-themes: *Striving to live an ordinary family life, yet feeling and living very different—*and sub-themes: *A life-changing situation, Always on guard*, and *Struggling to let go*.

### Striving to live an ordinary family life, yet feeling and living very different

Parenting a child with T1D aged 1 to 7 years was a challenge indeed, particularly when the child could not communicate clearly how he/she felt, as he/she did not quite understand what was going on. Parents described a balance between always being on guard and in a state of preparedness, and at the same time trying hard to let go of constant worries, even if it was for a short while. Striving to live like a “normal” family was a core part of their stories, bound up with and contrasted to what was experienced as a new “normality” in the lives they were now living. As time was passing, taking part in social events and activities with other families was essential for them, yet they felt they lived profoundly differently from other families. It was impossible to take no notice of their child´s diabetes, and not to see themselves as “therapists.” The condition meant always taking precautions and actions to ensure the child´s blood glucose status remained stable, sometimes also involving others, which could be challenging. Enough and correct food, having food available at all times and everywhere, haunted their minds. It was hard for them to be fully present in social situations, with others and also when together with their own child. Some parents even experienced that the diabetes came between their beloved child and themselves. They felt almost prevented from seeing the child on his/her own terms. The diabetes tended to take over. They expressed a need for more knowledge and support concerning the upbringing of a child with TD1.

#### A life-changing situation

It took quite a long time before the parents experienced that they downed that the situation was changed forever, and it had changed their role as parents. It was hard to accept the reality of their child’s chronic condition, and they felt overwhelmed by the new situation. The parents described that at first they did not understand what was going on, even though they had been worried about the child´s health prior to the diagnosis. They had to get familiar with the new situation, which was challenging. They had to learn about the disease, and how to handle it, and they had to learn quickly. The establishment of strict routines concerning the regulation of blood glucose was crucial, and they had to work together in order to manage that. Nevertheless, they experienced much stress in daily life, practically as well as emotionally, and involving existential concerns. Both mothers and fathers talked about a period of grief and sorrow, which took time to work through, and that was still going on. Even if the situation was no longer experienced to be as dramatic as in the beginning, feelings of despair could easily arise. One mother exclaimed:

But as time goes by there is not the same drama about the whole thing, but I still feel the despair and the loneliness when I let it come to me. (Mother child 5)

After about 6 months to 1 year, however, the parents were more familiar with the situation. When looking back in time, they felt they had been very distressed at the time around diagnosis. They talked about the imperative of learning very much, very quickly.

But then there is a steep learning curve, something I have never experienced the like of, one only has to hang in there. (Mother child 5)

Even if some of the frustration eased over time, they still thought a lot about the quality of their child’s childhood. What consequences for the child could there be when everyday practices and habits would consistently consist of thinking about regulating the blood glucose? Worries were expressed about long-term medical complications for a child diagnosed with T1D so young. What if they failed to regulate the blood glucose properly? The future was intertwined with existential concerns about the child and the family. In this respect, acquiring more knowledge about diabetes was a double-edged sword.

I have read too much about the consequences of long-term high blood glucose levels, and in those periods I feel stressed. I get confused. The family is like a health institution working to ensure that all ends well. Nothing else happens in our family. (Father child 7)

Nevertheless, parents were concerned about keeping their worries under control, as they were very busy taking some control of the changed situation by establishing strict routines concerning the regulation of the child’s blood glucose status. What kind of food, how much food, how often to eat etc. occupied their minds. In this situation, parents also reacted to the situation by doing practical tasks. Fathers for instance had to wash the car, work in the garden and so on.. Mothers tended to be more emotional in their expressions, and struggled with feelings of guilt; had they done something wrong during the pregnancy? A strong need for explanation was expressed:

I really wanted an explanation of this. Now I realise there is no explanation, I realise that more now than then, for then I was so desperate to find an explanation [when the child was diagnosed]. I was helped on track by being given explanations, at least I had not done anything wrong. (Mother child 5)

In this new situation, it was a balancing act to be parents. They wanted to raise the child on his/her own terms at the same time as taking care of the T1D treatment. As time was passing, parents understood the symptoms better, and gained more knowledge about how to regulate the blood glucose. Hence, they became better prepared to interpret what was going on. Nevertheless, parenting a child with diabetes at this age involved continuous challenges, as the child did not have an understanding of the symptoms and the consequences of not eating regularly. To find the balance in everyday life indicated to support each other as parents. For instance, they had to discuss their experiences and judgements, and make plans for the next night and day about insulin doses and food. However, the parents also expressed a kind of loneliness, as they had to work through this new situation alone as well. The parents described it like an experience of loss. Their child had been deprived of a life without worries about his/her health at such a young age. The seriousness of the disease, and the imperative of always acting on the premises of the blood glucose status disturbed the relationship with their child to some extent.

It feels as if the diabetes has come between me and my child, and to me that was kind of a feeling of loss. I longed to stay in the mother´s role. We had numerous conversations over this, the father and I, to focus on what happens here and now. (Mother child 7)

#### Always on guard

To be parents to a child at this age with T1D, indicated always to be on guard, always be forewarned and in a state of bodily and mentally readiness, just in case. To be in this on guard position over time implied challenges emotionally as well as challenges to their physical condition. Being the prime caregivers and facilitators all through day and night, and always being in need of control had its costs. Over time, it meant lack of rest, and a feeling of being constantly exhausted. Thoughts about the child’s blood glucose status were there all the time, the first thing in the morning, the last thing in the evening, and even through the night. One of the participants put it this way:

Diabetes is an underrated condition, you look healthy—nothing is visible. To be parents in this situation implies that you live in a state of perpetual attention. (Mother child 7)

Parents had to cooperate very concretely to regulate the blood glucose and other activities during the day, which could imply tensing up a bit. One father said:

My wife and I cooperate well, we feel that ourselves. We have had our, what shall I say,…. near fights where we have accused each other like, why did you forget this, don´t you know that this food contains lots of carbohydrates—yes, we have had a few of those. (Father child 8)

They were exhausted, but not necessarily at the same time. It was important that they gave each other room and time to rest. They also had to work on respecting each other’s judgements, and take for granted that both were doing their very best for the child.

A few weeks after the child was diagnosed, parents normally went back to their jobs, and the child returned to the kindergarten. That required that the staff have knowledge about how to take care of a child with T1D. The parents had the responsibility for giving this information to the staff. This challenge materialised at the same time as they were still being challenged to find their own new role as parents of a child with a chronic condition. That was felt to be an overwhelming challenge. A complicating factor was also the high turnover among the staff. The parents said there were frequent calls from the kindergarten, where staff asked repeatedly about concrete assessments and actions to take according to blood glucose status. The parents talked about leaving home or work, driving to the kindergarten and taking care of the child themselves. They understood however that it was a very great responsibility for the staff, especially when the child had unstable blood glucose values.

I really think about this all the time, in the way that I always must be available for phone calls from the kindergarten. (Father child 6)

The staff simply did not have sufficient experience and knowledge. Still, sometimes the parents also felt provoked by the staff’s lack of using knowledge that the parents had tried to provide.

One day I came to pick her up and her blood glucose was low. She was sitting there with a dry slice of bread, which she did not want. I was irritated and asked if they did not have any juice. In my opinion that is the kind of thing they should think of themselves. Then I can get cross and say things I may regret. (Father child 3)

Every third month, the parents accompanied their child to a consultation at a doctor’s office. The ultimate goal was for the child to have registered an acceptable HbA1c value. Often this was the case. Always to be on guard on the premises of their child’s health, seemed to give good results, but exhausted the mothers and fathers. Again and again, the parents gave examples of feeling haunted by thoughts about regulating the child’s blood glucose, when in charge of this themselves, and when others had to take responsibility.

…must monitor her symptoms all the time and be ready to take action. Things may change in minutes. All of this is difficult to cope with and requires adult persons near to observe what is going on. (Mother child 2)

They experienced that the relation to their child was changed. But not only that; the relation between the parents themselves was changed too.

As mother and father, we live with constant attention directed at the diabetes condition, but we have now acquired a higher grade of competence to tackle the challenges springing from diabetes. To live in this situation implies that the condition fills up our everyday life, and that is the way it has to be. (Mother/Father child 4)

#### Struggling to let go

Reducing social contact for some time after the diagnosis was part of becoming familiar with the new situation. The parents spent time and energy on learning to take care of the child during incidents of scary blood glucose fluctuations and regarding what to eat. When they became better acquainted with the situation, they were challenged to let others, such as family members or friends, take responsibility for the child, and not only in the places where the child had to stay on a daily basis. They emphasised that it was important that the focus on diabetes did not haunt them all the time, but it was very hard to let it go. When they managed to let go, and experienced that it went well, it was encouraging.

I discover there is a price to handing over the reins to others, yes there is. But the more we let friends and family help, and I see it working well, the easier it gets. (Mother child 5)

It was important for them to be a “normal” family, and letting their child stay in the care of others for a few hours was part of that. Their new way of living as regards health concerns and routines was described as their new normality. To let others take responsibility for their child still did not come easily, but they struggled to make this part of their “normality.” They wanted their child to live like other children, and this was an important part of that.

We have told our friends and the parents of the children’s friends that we want the kids to be treated like everybody else and be invited to children´s parties. We, as parents, take the responsibility for measurements and dosages in cooperation with our children. (Father child 8)

During childhood, it is important to establish social competence and friendship. Normally, children like to visit each other’s families. In situations where this issue came up, the parents thought about the knowledge the parents of the child´s friend had to posit and the actions they need to take if something happened. They were unsure about how much they could expect from other children´s parents.

She has followed a kindergarten girlfriend home, and this is challenging, as she cannot stay long because the mother knows nothing about diabetes. I wanted to offer to teach the mother but my husband thought this was too much… .now the girls stay with each other without problems. (Mother child 1)

Planning every activity, taking precautions about food, measuring insulin etc. were part of their daily life, and acquired a kind of familiarity as time went by. They expressed a need for social contact with other families just as a family, and wanted to re-establish contact with the families of friends of their children. However, when spending time with other families, they easily felt different. This difference became very visible when they were forced to educate parents of other children about the precautions they had to take or make these visible. The situation described below highlights this experience very well:

But when we and our friends were going on a holiday for a full week things happened. Disparities in the amounts of candy for the children made the mother of the friends feel that with so much consideration for our kids, her kids should also get what they wanted… This hurt more than I would have thought possible. (Mother child 3)

To let go implied leaving their child in others’ care, and trust them. It also implied letting go of the fear of being different in situations with other families, where their child’s diabetes became visible. One could say that the cost of being normal implied accepting being different.

To just be spouses and together as adults, without being haunted by their child’s diabetes, was important. That meant spending time together on their own, and allowing others to take responsibility. Diabetes is a lifelong condition, and the parents had to take care of themselves also, and take the offers of babysitting they received:

We are reluctant to go away and leave things to others, really think we are similar in this respect, she (the mother) and I. We have been on one trip by ourselves since this happened, and we have talked about it, about having to take care of ourselves as well. (Father child 5)

Some had family and good friends around in whom they trusted, but anyway it was demanding to leave the child in the custody of others. The necessity for many preparations before they could leave the responsibility to other persons, and many worries when they were absent were expressed:

There are family and friends ready when we need time for ourselves. But it is difficult to let go, and we think about all the considerations we must make all the time. It is difficult for us to leave this responsibility to others. (Mother child 7)

## Discussion

This study gives insight into mothers and fathers lived experiences of having a child with T1D. We will reflect on the core findings: *Striving to live an ordinary family life, yet feeling and living very different* **—**and sub-themes: *A life-changing situation, Always on guard*, and *Struggling to let go* considering existential dimensions involved; lived body, lived time, lived space and lived relations to others (Van Manen, 1997, p.106). This is in accordance with the phenomenological anchoring of the study. In so doing, we will also refer to previous research.

The parents described a life-changing situation when the child was diagnosed T1D, and it took quite a long time before the parents understood that the situation had changed forever. They were simply thrown into being responsible for the treatment of the child, day and night, which demanded the immediate establishment of new routines and the building of knowledge about monitoring the child´s blood glucose. Quite early in the new situation, the parents had to go back to work, and the child to the kindergarten. The parents were also responsible for teaching and guiding the staff, often at a distance. Frequent calls from the kindergarten occurred during their working hours, meaning there was no room to let go of the responsibility. The life-threatening character of the condition was an immense burden on their shoulders. A lot of stress was involved, and feelings of vulnerability. Having a child with T1D represented a disruption to the whole family´s life, and the parents` own needs were put completely on hold. In phenomenological literature, being struck by chronic and/or serious disease is described as a disruption, existential in character, in a person´s life (Carel, 2008; Svenaeus, 2005), and this goes for the whole nuclear family if one of its members falls chronically and seriously ill.

Previous studies have also described the situation as a family disruption from the parents’ perspective, where new family patterns and routines had to be established (Smaldone & Ritholz, 2011; Wennick & Hallström, 2007; Whittemore et al., 2012). Parents reported that they were working hard to establish as normal a life as possible for their child, implying adjustment to daily diabetes management tasks, and becoming more confident in their ability to care for the child over time (Whittemore et al., 2012). This is also in line with findings in this study. However, our study also points to parents’ experiences of the impossibility of being fully confident in the task of regulating the child’s blood glucose. In Lowes and Lyne (2000), the life-changing character of the situation of having a child with T1D was also underscored. Parents were interviewed at the first week, 4 months and 12 months after the diagnosis, and it was found that it took about 12 months to find a new way of living. According to our findings, this is more complicated. Even though parents were on their way to find a new “normality” of daily life for the family, they were still always on guard in case of “emergency” as to the child’s blood glucose monitoring, and they were at the same time struggling to let go of control, even for moments when the child was out of their immediate radius. This state of alertness, which was described by the parents in our study, was present up to 6 years after the diagnosis was a fact (from 1 to 6 years, see Table I). In another study (Streisand et al., 2008), parents reported feelings of isolation, and fear and doubt about being able to regulate the child’s blood-glucose level optimally pervaded these parents’ experiences. This may have some parallels to findings in our study about parents finding themselves in a constant state of alertness, fearing the grave consequences of not being able to regulate the child’s blood-glucose level properly, which also implied isolation. In our study, however, active efforts to break the isolation, and let go of control, were also described. It seems important to understand that processes related to actively managing the imperative of being responsible for the regulation of the child’s blood-glucose level, both implies the establishment of routines and increased mastering over time, and at the same time always fearing the possibility of not managing the daily demands of the condition. The child’s health and life itself is always at stake. Our study underscores these “opposing” reactions.

We will deepen the understanding of the parents’ experiences of their situation by highlighting how deeply interwoven changes in the existential dimensions are in the essential theme and its sub-themes, which in our study represent the meaning structure of the phenomenon of parenting a pre-school child with T1D. To understand more fully, what was at stake for the parents, we have to look at the parent—child relation itself. The parent—child relation is a special lived relation; symbiotic in character, and based in the responsibility for upholding life itself so to speak (Van Manen, 1997). This special, in our case, changed relation is at the heart of the changes in the existential dimensions of the parents’ life world.

At the time of the interviews, the parents in our study negotiated how to act in order for the child—and themselves—to live as normally as possible. Parents described always being on guard, in a state of readiness “just in case,” and were still trying to persuade themselves about the necessity of letting go every now and then. Van Manen said: “….*parents and children are of one flesh, in the physical holding and parental embrace we know our child in a profoundly symbiotic way*.” Merleau-Ponty, Landes, Carman, and Lefort (2012) notion of inter-subjectivity as inter-corporeally constituted is van Manen´s point of departure, with the relationship between parents and child being a prime example. Merleau-Ponty et al.’s (2012)claims that there is a mutuality between the lived body and self, the lived body and others, the lived body and world, meaning that lived experience is incorporated and expressed, for instance in habitual ways of reacting and relating in everyday life. The state of being constantly on guard, ready to act, and deeply worried seemed to be incorporated in the parents as a way of habitually relating to the child. Accordingly, lived bodies of parenting a small child were described as being in a state of alertness and worry, and no room for relaxation. The child´s health and whole future was at stake, and the interpersonal space they shared in the family seemed to be permeated by that. The young age of the child may also be an issue of importance here. As mentioned above, always being on guard seemed to be a condition involving the whole person’s way of relating and reacting, and was indeed very energy consuming. Parents spoke about being tired and exhausted most of the time. The seriousness of the responsibility they were carrying on their shoulders, all the practical tasks they had to remember and carry out, as well as information to the staff in the kindergarten and so on, point to increased intensity of the lived here and now. In the beginning, it seemed to be more than enough to keep up and keep going, not being able to plan for more than today, tonight and tomorrow, but certainly being very much worried about their child’s future. In this situation, lived time seemed to shrink to here and now. When negotiating about letting go later on, they were more able to stretch forward towards a “more normal” future. But, as one father expressed it, acting as if they were a whole “health enterprise” could still be the case.

They were also worried about the condition coming between themselves and their child, disturbing the relationship in profound ways. In other words, they experienced that the very relation to their child itself was at risk. It was in this situation that parents also talked about the necessity of negotiating with themselves about the courage to let go and give room for less control and some spontaneity, approaching living like an “ordinary” family. They had to take measures in order to re-establish a way of relating to the child that gave room for him/her to develop on his/her own terms. Lived relations within the family, and with others around them, changed profoundly when the child fell ill, and had to be re-negotiated. In this respect, they were concerned about giving the child the ability to establish friendships through visiting and taking part in activities with other families, and not always being present themselves. They felt it was important, but also very frightening. To widen the child’s social space was hard indeed, but at the same time felt as a necessity.

Previous studies have touched upon some of these aspects. Whittemore et al. (2012) found that parents expressed feelings of loss of spontaneity and freedom. In a study by Hatton, Canam, Thome, and Hughes (1995) mothers of infants and toddlers with diabetes reported a diminished bond and a loss of an ideal relationship with their child. Sullivan-Bolyai et al. (2006) interviewed fathers of children with T1D, and the fathers described an underlying sadness at the same time as they felt a great responsibility to be strong and to support their partners (the mothers). In the study of Smaldone and Ritholz (2011) on older children with T1D (mid. 11 years), parents realised that their child needed to have a sense of being capable and independent, but also experienced for example sleepovers to be anxiety-provoking because parents were unsure about their child`s safety in another`s home. In a Swedish study, parents of children with T1D (<18 years of age) reported significantly more symptoms of burnout than the parents in a control group (Lindström, Åman, & Norberg, 2010). Our study shows in more depth than previous research what is at stake from the parent’s perspective, and how the new situation “sits” in their bodily emotional ways of relating and reacting.

### Limitations

We were able to identify an essential theme concerning parenting a small child with T1D, with sub-themes describing interconnected aspects of the essential theme. As such, the material was rich enough to reach a level of abstraction that goes beyond a mere thematic analysis, and contributes to the understanding of the phenomenon of parenting a small child with T1D. However, the meaning structure of parenting a small child with T1D identified in our study is embedded in the context of the participants taking part (Wertz, 2011). Further studies of parents´ lived experience may enrich and expand the understanding.

## Conclusion

Our findings contribute to filling the gap in the knowledge about the lived experience of being parents of younger children with diabetes. Parents with a child with a newly diagnosed chronic condition felt overwhelmed by the new situation. When the situation was more settled, they strove to live like a “normal” family, yet feeling and living very differently. Our study underscores that parenting a small child with T1D is extremely demanding. Support from health care professionals at outpatient clinics primarily concerns obtaining knowledge about the condition and handling practical tasks, which is necessary. However, our study points to the need for support in the handling of emotional reactions as well.
